# Functional Analysis of MsepOR13 in the Oriental Armyworm *Mythimna separata* (Walker)

**DOI:** 10.3389/fphys.2019.00367

**Published:** 2019-04-09

**Authors:** Kunpeng Zhang, Yilu Feng, Lixiao Du, Shanshan Gao, Hang Yan, Kun Li, Nana Liu, Junxiang Wu, Guirong Wang

**Affiliations:** ^1^State Key Laboratory of Crop Stress, Northwest A&F University, Yangling, China; ^2^College of Biology and Food Engineering, Anyang Institute of Technology, Anyang, China; ^3^State Key Laboratory for Biology of Plant Diseases and Insect Pests, Institute of Plant Protection, Chinese Academy of Agricultural Sciences, Beijing, China

**Keywords:** *Mythimna separata*, odorant receptor, eugenol, *Xenopus* oocytes, odorant tuning

## Abstract

Olfaction in insects has a critical role in recognizing the host, finding food, and choosing mating partners, as well as avoiding predators. Odorant receptors (ORs), which are housed in the dendritic membrane of sensory neurons and extended into the lymph of sensilla on insect antennae, are participating in the detection of volatile compounds in insects. In the present study, we identified an OR gene, named *MsepOR13*, in the oriental armyworm *Mythimna separata* (Walker). Quantitative real-time polymerase chain reaction revealed that *MsepOR13* was expressed mainly in the antennae of male and female moths. In *in vitro* heterologous expression experiments, MsepOR13 was widely tuned to 32 of the 67 different compounds tested. Furthermore, MsepOR13 responded to eugenol at a low concentration of 10^-9^ M, with an EC50 value of 3.91 × 10^-6^ M. The high sensitivity suggests an important role for the OR13 gene in the moth olfactory system.

## Introduction

Chemoreception of odorants in the environment is critically important for the survival of insects. During evolution, insects have evolved a powerful sense of olfaction to locate hosts and mating partners, identify oviposition sites, discriminate toxic food, and escape predators (Schneider, 1969; Bruce et al., 2005; Bruyne and Baker, 2008; Hansson and Stensmyr, 2011; Gadenne et al., 2016), as they are surrounded by various chemical compounds emitted from conspecifics, predators, and host plants (Bentley and Day, 1989; Schneider, 1992; Hansson and Stensmyr, 2011). These odorants are diffused to the surface on olfactory appendages, which mainly consisting of antennae and maxillary palps (Steinbrecht, 1997), and enter the lymph through pores of the sensilla, which are hair-like structures. Odorant molecules interact with odorant-binding proteins (OBPs) in the sensilla lymph and are transferred toward the dendrites of olfactory sensory neurons (OSNs), where odorant receptors (ORs) are expressed. Activation of ORs leads to chemical information being transduced to electrical signals, which are conveyed to the antennal lobe and finally decoded by the insect brain (Vogt, 2003; Leal, 2013).

Owing to the availability of the *Drosophila melanogaster* genome sequence, the first insect OR was identified in *D. melanogaster* based on the homology of OR sequences in vertebrates and nematodes and the restricted expression of these genes in olfactory tissues (Clyne et al., 1999; Vosshall et al., 1999). Compared with G-protein coupled receptors (GPCRs), insect ORs have the opposite membrane topology, with their N-terminus inside and their C-terminus outside the cell; this is an inverse membrane topology to that found in vertebrate ORs (Buck and Axel, 1991; Benton et al., 2006; Fleischer et al., 2017; Butterwick et al., 2018). It is now generally accepted that insect ORs transduce chemical signals by forming heteromeric complexes with an OR co-receptor (Orco) that operate as non-selective cation channels (Koji et al., 2008; Wicher et al., 2008).

In recent decades, with progress in sequencing technology and bioinformatics tools, numerous ORs have been reported in many species from various insect orders, including Lepidoptera, Diptera, Hymenoptera, Coleoptera, Hemiptera, Orthoptera, and Phthiraptera. The number of OR genes varies considerably among insect species. For example, there are 65 ORs in *Helicoverpa armigera* (Liu et al., 2012; Zhang et al., 2015a) and 62 ORs in *Mythimna separata* (Du et al., 2018), based on antennal transcriptomic analysis, whereas 163 ORs have been obtained from the genome of *Apis mellifera* (Robertson and Wanner, 2006) and 256 ORs have been identified in the genome of *Tribolium castaneum* (Engsontia et al., 2008). The variation in number of ORs between insects is assumed to correlate with evolutionary adaption to certain ecological and physiological demands (Fleischer et al., 2017).

Although increasing numbers of OR genes have been identified during recent decades, the functional characterization of the encoded proteins lags significantly behind. Heterologous *in vitro* expression systems, such as cultured cell lines and *Xenopus* oocytes, and *in vivo* expression systems, such as the “empty neuron system” of *Drosophila*, have been successfully established for functional analysis of insect ORs (Dobritsa et al., 2003; Gonzalez et al., 2016; Wang et al., 2016). These systems have been applied for functional characterization of both pheromone and non-pheromone receptors in several species, including *D. melanogaster* (Hallem et al., 2004; Kreher et al., 2005; Hallem et al., 2006), *Anopheles gambiae* (Lu et al., 2007; Carey et al., 2010; Wang et al., 2010), *B. mori* (Sakurai et al., 2004, 2011; Nakagawa et al., 2005; Grosse-Wilde et al., 2011), *Heliothis virescens* (Ewald et al., 2010; Wang et al., 2011), *Ostrinia nubilalis* (Wanner et al., 2010; Yuji et al., 2011; Leary et al., 2012), *O. furnacalis* (Miura et al., 2010; Liu W. et al., 2018), *Spodoptera littoralis* (de Fouchier et al., 2017), *Cydia pomonella* (Bengtsson et al., 2014; Gonzalez et al., 2015; Cattaneo et al., 2017), *H. armigera* (Liu Y. et al., 2013; Cao et al., 2016; Chang et al., 2016; Di et al., 2017)*, H. assulta* (Chang et al., 2016; Cui et al., 2018), *Plutella xylostella* (Sun et al., 2013; Liu Y. et al., 2018), *S. exigua* (Liu C. et al., 2013; Liu et al., 2014), and *S. litura* (Zhang et al., 2015b).

The oriental armyworm *M. separata* (Walker) (Lepidoptera: Noctuidae) is an economically important and common lepidopteran pest, which is widely distributed in eastern Asia and Australia, and attacks many crop plants including maize, sorghum, and rice. *M. separata* migrates long distances, resulting in widespread incidence, which can lead to complete crop loss (Sharma and Davies, 1983; Jiang et al., 2011). In recent years, *M. separata* has been observed in many regions of China and poses a severe threat to corn production. In order to control this pest, high doses of insecticides are often applied; however, this has some negative effects, including environmental pollution, insect resistance, and harm to non-target organisms (Lv et al., 2014; Duan et al., 2017). Outbreaks of *M. separata* represent a great challenge in crop protection worldwide (Liu et al., 2017).

Compared with the use of chemical pesticides, olfactory-baited trapping is an effective and environmentally friendly method to manage *M. separata*. The sex pheromone of *M. separata* has been used in this way (Wei, 1985; Zhu et al., 1987), but the effect was unsatisfactory for unknown reasons. *Pterocarya stenoptera* and *Salix babylonica* are also used to attract *M. separata* in the field (Lihuang et al., 2017), although the mechanism of attraction is unknown. In previous work, we identified the ORs in *M. separata* using transcriptomic analysis (Du et al., 2018), but no study on their function has been reported except for MsepOR1, responding to the major sex pheromone compound Z11-16:Ac (Mitsuno et al., 2010). In the present study, we cloned an OR, named *MsepOR13*, in *M. separata* and analyzed the expression patterns in different tissues of both sexes by quantitative real-time polymerase chain reaction (qRT-PCR). Functional analysis was completed using *in vitro* expression in a *Xenopus* oocyte system with two-electrode, voltage-clamp physiological recordings.

## Materials and Methods

### Insect Rearing

The *M. separata* colony, maintained at the laboratory of Henan Agricultural University, Zhengzhou, China, was reared on an artificial diet at 28 ± 1°C, 70% ± 5% relative humidity, and a 14 h:10 h light:dark (L:D) photoperiod. Adult male and female moths were fed with 10% sugar solution.

### RNA Extraction and cDNA Synthesis

Male and female antennae, proboscises, labial palps, and legs (a mixture of female and male) of virgin male or female individuals were collected 3 days after eclosion, immediately frozen in liquid nitrogen, and stored at -70°C until RNA extraction. Total RNA of 20 adult male or female moths was isolated using TRIzol reagent (Invitrogen, Carlsbad, CA, United States) following the manufacturer’s instructions. Total RNA was dissolved in RNase-free water and gel electrophoresis was performed to assess its integrity. RNA concentration and purity were determined on a Nanodrop ND-2000 spectrophotometer (NanoDrop products, Wilmington, DE, United States).

First, total RNA was treated with DNase I (Fermentas, Glen Burnie, MD, United States) for 30 min at 37°C to remove residual gDNA. Then, 1 μg total RNA was used to synthesize single-stranded cDNA as per the First Strand cDNA Synthesis Kit (Fermentas) manufacturer’s instructions. The cDNA of antennal samples was used as a template to clone the *MsepOR13* gene. The cDNA samples isolated from different female and male tissue types were used as templates for RT-qPCR.

### Cloning of *MsepOR13* Gene From *M. separata*

The sequence of *MsepOR13* was identified in *M. separata* by transcriptomic analysis (Du et al., 2018). Specific primers were designed by Primer 5.0 (PREMIER Biosoft International, Palo Alto, CA, United States) to clone the full-length sequence of MsepOR13 (Table 1). Antennal cDNA from female and male moths was used to amplify the full-length sequence of *MsepOR13* using primeSTAR HS (Premix) (TaKaRa, Dalian, China). PCR reactions of 50 μL contained 25 μL 2×primeSTAR HS (Premix), 1.5 μL sense and anti-sense primers (10 μM), 2 μL cDNA, and 20 μL double-distilled H_2_O. Reactions were carried out under the following conditions: 95°C for 3 min; 35 cycles of 95°C for 30 s, 57°C for 30 s, and 72°C for 1 min; and 72°C for 10 min; before being held at 16°C. PCR products were analyzed on a 1.5% agarose gel and the sequence was sub-cloned to the vector pEASY-Blunt (TransGene, Beijing, China). The sequencing was completed in Sangon Biotech, Shanghai, China.

**Table 1:** Primers’ sequence in this study.

Primers	Sequences 5′–3′	Purpose
*MsepOR13*-F	ATGGCGGATATTCCAACGG	Gene cloning
*MsepOR13*-R	TTAACGATTCAAAAATGTAA ACAAGGT	
*MsepOrco*-F	ATGATGACCAAAGTGAAGGC	
*MsepOrco*-R	TTACTTGAGTTGCACCAACAC	
*MsepOR13*-qF	GGAAGCAGCGTGTCAATGTT	qPCR
*MsepOR13*-qR	AGGTCTCGGGAAGTTCTCCA	
*MsepRPS3*-qF	AATGAGTTCTTGACCAGGGAG	
*MsepRPS3*-qR	GTGTCCTCGTCGCCATAAT	
*MsepOR13*-A	TCAgggcccGCCACCATGGCG GATATTCCAACGG	cRNA synthesizing
*MsepOR13*-S	TCAgcggccgcCTTAACGAT TCAAAAATGTAAACAAGGT	
*MsepOrco*-A	TCAgggcccGCCACCATGAT GACCAAAGTGAAGGC	
*MsepOrco*-S	TCAgcggccgcTTACTTGAG TTGCACCAACAC	


### Sequence Analysis

The amino acid sequence of MsepOR13 was determined using the ExPASy-Translate tool^1^. The sequence was aligned with ORs from *Peridroma saucia* (PsauOR, GenBank: AVF19631.1), *Athetis lepigone* (AlepOR19, GenBank: AOE48024.1), and *Athetis dissimilis* (AdisOR31, GenBank: ALM26220.1) using DNAMAN version 8 (Lynnon LLC, San Ramon, CA, United States).

### Tissue Expression Profile of *MsepOR13*

Quantitative polymerase chain reaction was performed to determine the expression of *MsepOR13.* Male and female antennae, proboscises, labial palps, and legs (a mixture of female and male) were collected from 3-day-old *M. separata* adults after eclosion. RNA extraction and cDNA synthesis were performed following the protocol described above. *MsepRPS3* was chosen as the reference gene. The primers are listed in Table 1. GoTaq qPCR Master Mix (Promega, Madison, WI, United States) was used for qPCR, and the reactions were carried out on an Applied Biosystems 7500 Fast Real-Time PCR System (ABI, Carlsbad, CA, United States). The reactions (20 μL) consisted of 10 μL GoTaq qPCR Master Mix, 0.8 μL gene primer (10 μM), 1 μL cDNA, and 7.4 μL RNase-free water. The reactions were carried out under the following conditions: 95°C for 2 min; 40 cycles of 95°C for 15 s, and 60°C for 50 s. Each qPCR reaction was performed in triplicate with three independent biological samples to check reproducibility. The melting curves were inspected to check the specificity of the primers, and the amplification efficiencies were calculated by the standard curve method. The efficiency of the primers for *MsepOR13* and *MsepRPS3* were 97 and 105%, respectively. *MsepOR13* relative expression levels were analyzed using the relative 2^-ΔΔCT^ quantitation method, where ΔC_T_ = C_T_ (*MsepOR13*) – C_T_ (*MsepRPS3*), ΔΔC_T_ = ΔC_T_ (different samples) – ΔC_T_ (legs (female and male mixture)). Statistical comparison of expression of *MsepOR13* was assessed using one-way nested analysis of variance (ANOVA), followed by least-significant difference (LSD) tests.

### MsepOR13 Expression in *Xenopus* Oocytes and Electrophysiological Recordings

The full-length *MsepOR13* was first cloned into a pEASY-Blunt vector and then ligated into a pT7Ts expression vector using primers containing *Apa I* (GGGCCC) and *Not I* (GCGGCCGC) sites. The expression vector was linearized using *Sma I* (CCCGGG) (Fermentas, Glen Burnie, MD, United States) and the cRNA was synthesized using an mMESSAGE mMACHINE T7 kit (Ambion, Austin, TX, United States). Mature healthy *Xenopus* oocytes (stages V–VII) were incubated with 2 mg/mL collagenase I in pH 7.6 washing buffer consisting of 96 mM NaCl, 2 mM KCl, 5 mM MgCl_2_, and 5 mM HEPES at room temperature for about 1 h until almost of them were separated a signal one. Then, 27.6 ng MsepOR13 cRNA and 27.6 ng MsepOrco cRNA were microinjected together into oocytes, and the oocytes were cultured in 1× Ringer’s buffer (washing buffer supplemented with 0.8 mM CaCl_2_, 5% dialyzed horse serum, 50 mg/mL tetracycline, 100 mg/mL streptomycin, and 550 mg/mL sodium pyruvate) for 4–7 days. The whole-cell currents of injected oocytes were recorded with an OC-725C oocyte clamp at a holding potential of -80 mV (Warner Instruments, Hamden, CT, United States), following previously described experimental procedures (Cui et al., 2018; Liu W. et al., 2018; Liu Y. et al., 2018). Oocytes were exposed to different compounds at 10^-4^ M for 15 s each, in a random order, with intervals between exposures that allowed the current to return to baseline. Dose–response curves were acquired from 10^-9^ to 10^-4^ M in ascending order of concentration. All data acquisition and analysis were carried out with Digidata 1440A and Pclamp10.0 (Axon Instruments, Inc., Union City, CA, United States), and dose–response data were analyzed using GraphPad Prism 5. Statistical comparison of responses to different odors of MsepOR13 was assessed using ANOVA, followed by LSD tests.

### Odorant Panel

Sixty-seven plant volatile compounds purchased from Sigma-Aldrich were used in this experiment (Table 2) and were classified into six groups: terpenoid, aromatic, alcohol, ester, aldehyde, and ketone. All compounds were dissolved in dimethyl sulfoxide (DMSO) at a concentration of 1 M as stock solutions. Before the experiments, the stock solutions were diluted in 1× Ringer’s buffer to working concentrations, and 1× Ringer’s buffer containing 0.1% DMSO was used as a negative control.

**Table 2:** Test odorants in functional analysis of MsepOR13 in *M. separate.*

Class	Odorant	CAS no.	Purity (%)	Class	Odorant	CAS no.	Purity (%)	Class	Odorant	CAS no.	Purity (%)
Terpenoid	β-Citronellol	106-22-9	95	Terpenoid	Cedrol	77-53-2	99	Aromatic	Ethyl benzoate	93-89-0	99
	Geraniol	106-24-1	98		β-Ionone	79-77-6	96		Butyl salicylate	2052-14-4	99
	(1S)-(–)-Verbenone	1196-01-6	93		Eucalyptol	13877-91-3	99		Methyl eugenol	93-15-2	98
	(S)-*cis*-Verbenol	18881-04-4	95		(R)-(–)-Piperitone	4573-50-6	94		δ-Decanolactone	705-86-2	98
	3,7-Dimethyl-3-octanol	78-69-3	98	Aromatic	Methyl benzoate	93-58-3	98	Alcohol	*cis*-3-Hexen-1-ol	928-96-1	98
	(1R)-(–)-Myrtenol	19894-97-4	95		4-Ethylbenzaldehyde	4748-78-1	99		*cis*-2-Hexen-1-ol	928-94-9	95
	(–)-*trans*-Pinocarveol	547-61-5	96		3-Vinylbenzaldehyde	19955-99-8	97		1-Heptanol	111-70-6	98
	(–)-Linalool	126-91-0	95		Benzaldehyde	100-52-7	99		1-Hexanol	111-27-3	98
	Linalool	78-70-6	97		4-Ethylacetophenone	937-30-4	97		*trans*-3-Hexen-1-ol	928-97-2	97
	Myrcene	123-35-3	95		Cinnamaldehyde	104-55-2	95		4-Hydroxy-4-methyl-2-pentanone	123-42-2	99
	(R)-(+)-Limonene	5989-27-5	97		Benzyl acetate	140-11-4	99		1-Octanol	111-87-5	98
	α-Pinene	80-56-8	98		Methyl salicylate	68917-75-9	99		*trans*-2-Hexen-1-ol	928-95-0	95
	(–)-β-Pinene	18172-67-3	99		2,6-Di-tert-butylphenol	128-39-2	99		1-Octen-3-ol	3391-86-4	98
	Camphene	79-92-5	95		Acetophenone	98-86-2	99	Ester	*cis*-3-Hexenyl acetate	3681-71-8	98
	(S)-(–)-Limonene	5989-54-8	95		Salicylaldehyde	90-02-8	98		*trans*-2-Hexenyl acetate	2497-18-9	98
	α-Terpinene	99-86-5	95		Methyl 2-methoxybenzoate	606-45-1	97		Geranyl acetate	105-87-3	97
	(–)-*trans*-Caryophyllene	87-44-5	98		Benzyl alcohol	100-51-6	99	Aldehyde	*trans*-2-Hexen-1-al	6728-26-3	95
	(–)-Caryophyllene oxide	1139-30-6	95		2-Phenylethanol	60-12-8	99		Heptanal	111-71-7	95
	Farnesene	502-61-4	98		4-Methoxybenzyl alcohol	105-13-5	98	Ketone	(±)-Camphor	76-22-2	98
	(1R)-(–)-Myrtenal	18486-69-6	97		Methyl phenylacetate	101-41-7	98		2-Pentadecanone	2345-28-0	95
	(±)-Citronellal	106-23-0	95		Eugenol	97-53-0	99		*cis*-Jasmone	488-10-8	94
	Ocimene	13877-91-3	90		Phenyl acetaldehyde	122-78-1	95				
	Nerolidol	40716-66-3	98		Methyl 4-hydroxybenzoate	99-76-3	99				


## Results

### Gene Cloning and Sequence Analysis of MsepOR13

**FIGURE 1:**
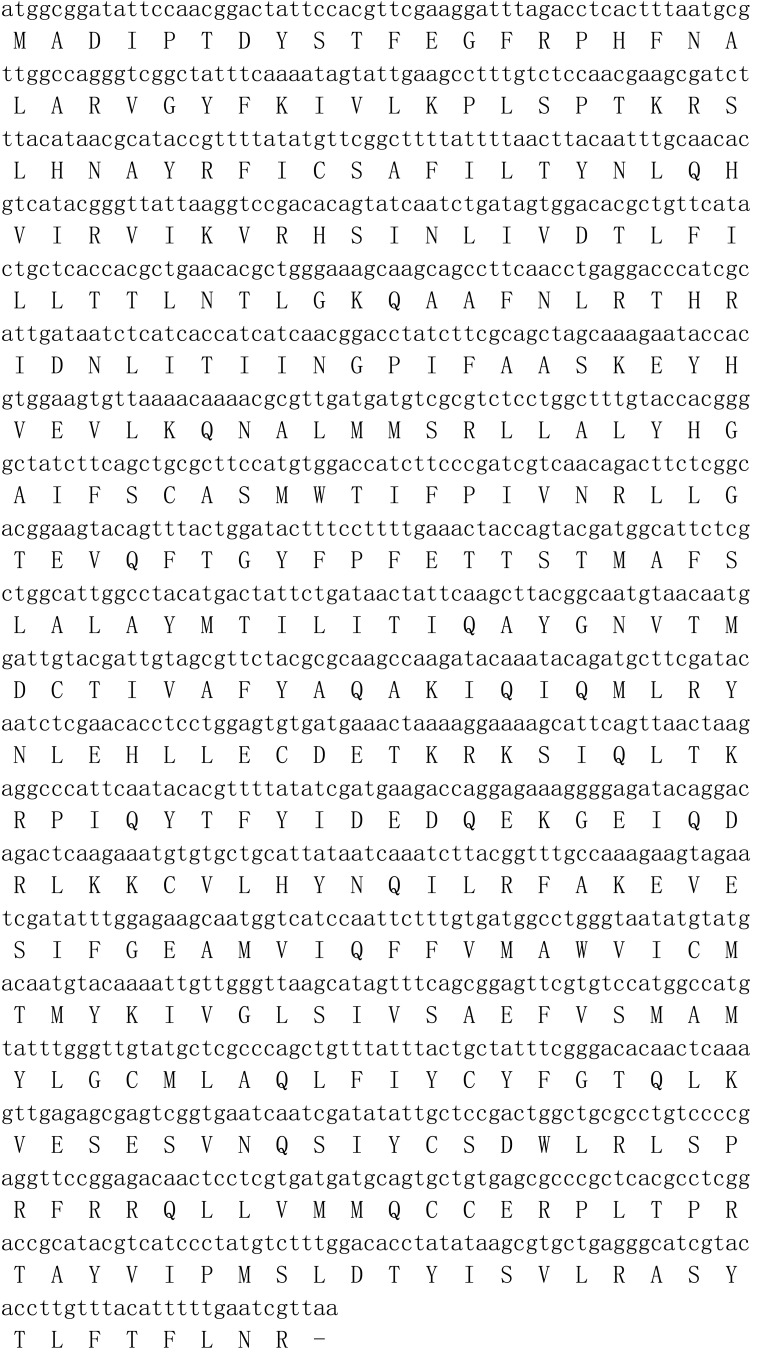
Nucleotide and amino acid sequences of the *MsepOR13* gene in *Mythimna separata*.

**FIGURE 2:**
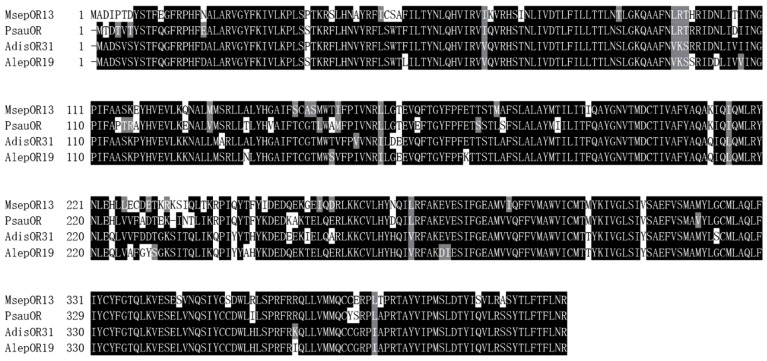
Alignment of the amino acid sequences of MsepOR13 to those of its homologs in other species. Amino acids identical in all sequences are marked with black shading. Numbers to the right refer to the position of the last residue in a line in each odorant receptor (OR) sequence. The horizontal lines indicate the position of predicted transmembrane domains.

Based on the transcriptome of *M. separata* (Du et al., 2018), we obtained the full-length sequence of *MsepOR13*. It contained 1227 bp, encoding 408 amino acids (Figure 1) Three amino acid sequences from *P. saucia* (PsauOR, GenBank Accession No. AVF19631.1), *A. lepigone* (AlepOR19, GenBank Accession No. AOE48024.1), and *A. dissimilis* (AdisOR31, GenBank Accession No. ALM26220.1) were aligned with MsepOR13 (Figure 2) and found to have 84, 81, and 83% identity, respectively.

### Tissue Expression Profiles of *MsepOR13*

**FIGURE 3:**
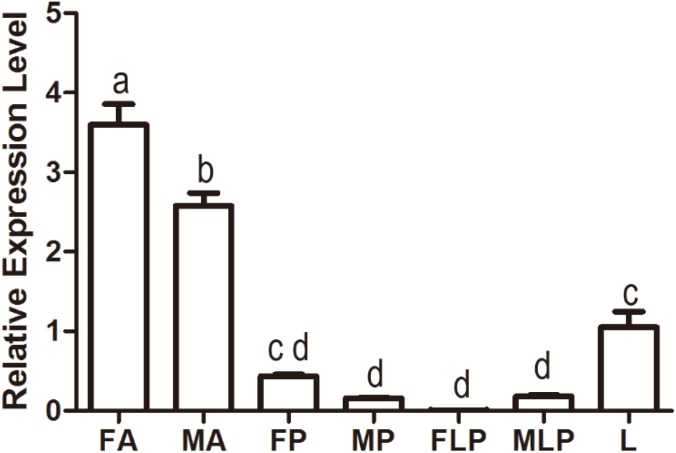
Tissue- and sex-specific expression of *MsepOR13* in *M. separata*. FA, female antennae; MA, male antennae; FP, female proboscis; MP, male proboscis; FLP, female labial palp; MLP, male labial palp; L, legs (both sexes mixed). Error bars represent the standard error; those labeled with different letters are significantly different (*p* < 0.05, ANOVA, LSD).

Quantitative polymerase chain reaction was carried out to evaluate the expression profile of *MsepOR13* in different tissues of both sexes in *M. separata*. The results showed that *MsepOR13* was mainly expressed in antennae compared with other tissues and exhibited much higher relative expression level in female antennae than male antennae (Figure 3). *MsepOR13* was less expressed in proboscis and labial palp in both sexes and there was no significant difference in the expression levels of *MespOR13* between leg (mixture of female and male moths) and female proboscis.

### Functional Characterization of MsepOR13 in the *Xenopus* Oocyte Expression System

**FIGURE 4:**
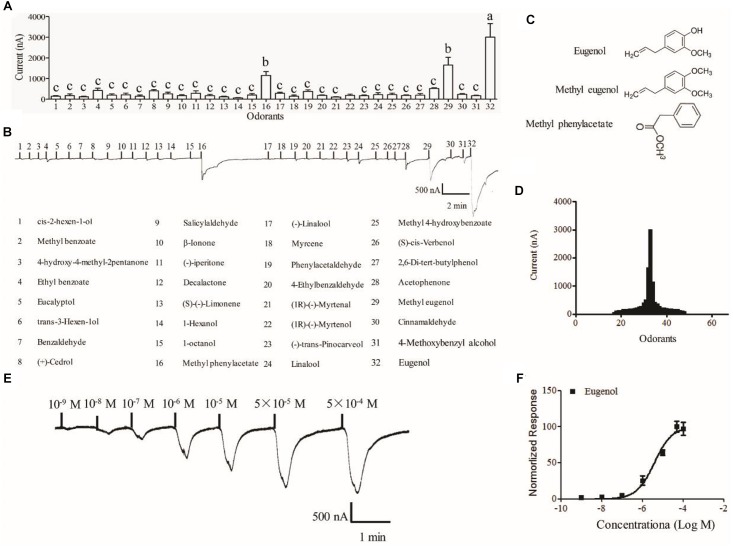
Functional response of *Xenopus oocytes*, with co-expressed MsepOR13/Orco, to volatile compounds. **(A)** Response profile of MsepOR13/Orco *Xenopus oocytes*. Error bars indicate standard error of the mean and bars labeled with different letters are significantly different (*p* < 0.001, ANOVA, LSD, *N* = 3–8); **(B)** responses of MsepOR13/Orco *Xenopus* oocytes to odorants in 10^-4^ M solution; **(C**) structure of the main ligands of MsepOR13. **(D)** Tuning curve for the MsepOR13 for an odorant panel comprising 67 odorants, arranged along the x-axis according to the strength of the response they elicit. The odorants that elicited the strongest responses were placed near the center of the distribution, while those that elicit the weakest responses were placed near the edges. **(E)** MsepOR13/Orco *Xenopus* oocytes stimulated with a range of eugenol concentrations. **(F)** Dose–response curve of MsepOR13/Orco *Xenopus* oocytes with eugenol. Responses were normalized by defining the maximal response as 100. The response value is given as mean ± standard error (*N* = 6).

The *Xenopus* oocyte expression system was used to identify candidate ligands for MespOR13. The cRNA of *MsepOR13* and *MespOrco* were co-injected into *Xenopus* oocytes, and responses to 67 compounds were recorded using a two-electrode voltage clamp. MsepOR13 was tuned to 32 odorants from all six classes and was most sensitive to eugenol, with responses of about 3011 nA (Figure 4A,B,D). In addition, methyl eugenol and methyl phenylacetate elicited the second strongest responses, of about 1655 and 1150 nA, respectively (Figure 4A,D). Interestingly, these three main legends shared similar structure, a benzene ring (Figure 4C). The other 29 odorants elicited the same response level. Acetophenone elicited a relatively higher response (523.3 nA) and 1-hexanol elicited the lowest response with an amplitude of 60 nA (Figure 4A,D). In the dose–response study, *Xenopus* oocyte co-expressing MsepOR13/MsepORco responded to 10^-9^ M of eugenol and the peak amplitude occurred at the concentration of 10^-5^ M (Figure 4E). The EC50 value of eugenol was 3.91 × 10^-6^ M (Figure 4F).

## Discussion

Detection of chemical odors in the environment is essential for the survival of insects. Accordingly, insects have evolved remarkable sensitive and discriminatory olfactory systems for locating hosts and food sources, identifying mating partners and oviposition sites, or escaping predators (Schneider, 1969; Hansson and Stensmyr, 2011; Gadenne et al., 2016). Previous studies have shown that ORs play an important part in the recognition of odorants and the process of chemo-electrical transduction (Leal, 2013; Wicher, 2014; Bohbot and Pitts, 2015). In this study, we cloned an OR gene, *MsepOR13*, in *M. separata*. The sequence contained 1227 bp, encoding 408 amino acids. As showed in the qPCR experiment, MsepOR13 exhibited female antennae-biased expression, which suggested that it might play a vital role in regulating female-specific behaviors, such as oviposition sites selection (Liu et al., 2015). Meanwhile, we found *MsepOR13* was also expressed in legs indicating that legs might assist insects to choose suitable oviposition sites. Previous studies found that female butterflies perceive oviposition stimulant by their foreleg tarsus and further determine the suitable feeding plant for larvae in *Papilio polytes* (Nakayama et al., 2003). Furtherly, 4 ORs were also identified by the legs transcriptome analysis in *Ectropis obliqua* (Ma et al., 2016), indicating that ORs expressed in legs was a ubiquitous phenomenon. During the past decade, the sex pheromone receptors have been well-deorphanized in many Lepidoptera species. However, the identification of ligands for the non-pheromone receptor ORs has significantly lagged behind, except for a few species such as *D. melanogaster* (Hallem et al., 2004; Kreher et al., 2005; Hallem et al., 2006), *A. gambiae* (Lu et al., 2007; Carey et al., 2010; Wang et al., 2010), and *S. littoralis* (Montagné et al., 2012; de Fouchier et al., 2017). In this study, MsepOR13 responded to 32 odorants and only three ligands elicited relative large response; this phenomenon was also found in studies of *S. littoralis* (de Fouchier et al., 2017) and *H. armigera* (Di et al., 2017). Narrowly tuned receptors are thought to be important in the detection of odors of high biological salience. In *D. melanogaster*, several ORs selectively responded to odors that are necessary and sufficient for vital behaviors such as avoiding toxic microbes and choosing oviposition sites (Stensmyr et al., 2012; Dweck et al., 2013, 2015; Ronderos et al., 2014). In mosquitoes, receptors that selectively respond to human emanations play a crucial part in host recognition and blood feeding (Hughes et al., 2010; Mcbride et al., 2014). Sex pheromone perception in moths also involves such specific pathways (Miura et al., 2010; Liu Y. et al., 2018). The homolog of MsepOR13 in *S. littoralis*, SlitOR31 shared 80% amino acid identity with MsepOR13. But SlitOR31 was narrowly tuned to eugenol, which is different from the function of MsepOR13. The difference of their function might relate with the different environment and the selective pressures they face.

In *M. separata*, the three main ligands containing a benzene ring were structurally similar; a similar phenomenon has been found in functional studies of ORs in *A. gambiae* ((Wang et al., 2010), *S. littoralis* (de Fouchier et al., 2017), and *H. armigera* (Di et al., 2017). Among all the ligands, eugenol activated the strongest response in MsepOR13/Orco *Xenopus* oocytes, and could response at a 10^-9^ M concentration, with an EC50 value of 3.91 × 10^-6^ M. Actually, MspeOR13 responding to eugenol showed the similar sensitivity with the reported pheromone receptors to sex pheromones (Liu C. et al., 2013; Chang et al., 2016; Liu W. et al., 2018; Liu Y. et al., 2018), suggesting that eugenol might be important to *M. separata*. It has been reported that eugenol can repel the *H. armigera* moth (Xu, 2004), and also repel *Populus yunnanensis* oviposition (Ma et al., 2016). In *Tribilium castaneum*, eugenol has apparently repellent activity toward adults and toxic effects on both larvae and adults (Han and Huang, 2009). However, in *Mamestra brassicae*, eugenol was found to attract larvae and moths (Yan, 2015). The functions of eugenol with respect to *M. separata* require further study, especially behavioral experiments, in order to develop environmentally friendly approaches to control this economically significant insect. Based on the high sensitivity of MsepOR13 to eugenol, we predict that MsepOR13 may have an important role in the reception of eugenol in *M. separata*; thus, its function could be further explored using the CRISPR-Cas9 system.

## Author Contributions

KZ and YF designed the experiments. HY, KL, and NL carried out the experiments. LD and SG analyzed the experimental results. JW and GW wrote the manuscript.

## Conflict of Interest Statement

The authors declare that the research was conducted in the absence of any commercial or financial relationships that could be construed as a potential conflict of interest.

## Footnotes

1 http://web.expasy.org/translate/
